# Prioritizing perioperative organ injury prevention: a call to action from China's county-level hospitals

**DOI:** 10.3389/fmed.2026.1758034

**Published:** 2026-01-22

**Authors:** Gang Li

**Affiliations:** Department of Urology Surgery, Mianzhu Hospital, West China Hospital, Sichuan University, Mianzhu, Sichuan, China

**Keywords:** county-level hospitals, global surgery, health systems strengthening, perioperative organ injury, preventive strategies, resource-limited settings

We read with great interest the landmark study by Kork et al., which comprehensively analyzed the impact of perioperative organ injury (POI) on morbidity and mortality in over 28 million surgical patients in Germany (1). This study underscores that POI is not merely a postoperative complication, but a significant public health burden of global magnitude, associated with a staggering 9-fold increase in the odds of death.

From the perspective of China's vast and tiered healthcare system, particularly through the lens of county-level hospitals which serve as the critical backbone of medical care for over half of China's population, this research resonates deeply and prompts urgent reflection and action.

## 1 The Chinese context: a larger scale with unique challenges

While the German data is compelling, the scale and setting in China are vastly different. China performs an estimated 45–50 million inpatient surgeries annually, with county-level hospitals undertaking a substantial proportion of these procedures (2–4). The burden of perioperative organ injury in China is significant even at a national level. For instance, large multicenter studies involving millions of hospitalized patients across diverse hospital tiers report adjusted incidences of acute kidney injury (AKI) as high as 11.6%, with associated in-hospital mortality ranging from 8.8% to 16.5% (5–7). It is crucial to note that these figures represent an average across hospitals of varying capabilities. In county-level hospitals, which often face constraints in diagnostic frequency, specialist support, and critical care resources, the true incidence of POI is likely under-recognized, and its impact on patient outcomes is potentially more severe than these national averages suggest. This gap underscores the urgent need for tailored preventive strategies within our unique healthcare landscape.

County-level hospitals, which we represent, bear the responsibility for a massive volume of basic and emergency surgeries. They serve an aging population with a high prevalence of comorbidities. The findings of Kork et al.—that patients with POI are older, have more comorbidities, and more often undergo emergency or high-risk surgery—perfectly describe a substantial portion of our patient population. However, our challenges are amplified:

### 1.1 Resource disparity

Unlike the well-resourced German hospitals, our institutions often face limitations in intensive care unit (ICU) beds, advanced monitoring equipment, and the availability of subspecialty consultants (e.g., nephrologists, neurologists) around the clock.

### 1.2 Prevention over rescue

The study highlights that Acute Kidney Injury (AKI) is the largest contributor to perioperative death. In our setting, where continuous renal replacement therapy (CRRT) capabilities may be limited, preventing AKI becomes infinitely more critical than treating its severe forms. Similarly, the extreme mortality associated with liver injury (68.7%) and ARDS (44.7%) makes their prevention a top priority, as successful rescue is often beyond our current capacities (1).

## 2 Re-evaluating “low mortality” injuries: the case of delirium

The study noted delirium had the “lowest” mortality (10.8%). However, from a functional outcomes and healthcare economics perspective, delirium is a colossal burden. It leads to prolonged hospital stays, increased nursing demands, and long-term cognitive decline. This places an immense strain on families and community healthcare resources (8). In our geriatric surgical population, preventing postoperative delirium through multidisciplinary, non-pharmacological bundles is a cost-effective and vital strategy that we are actively promoting.

## 3 A proposed framework for action in China

Inspired by this research, we propose a three-tiered strategy tailored for China's county hospitals:

### 3.1 Primary prevention (preoperative)

Enhance preoperative optimization clinics. Aggressively manage anemia (9–11), optimize cardiorenal function (12, 13), and implement frailty screening (14). For high-risk patients, consider transferring to higher-tier hospitals before surgery, not after complications occur.

### 3.2 Secondary prevention (intraoperative)

Mandate and standardize goal-directed fluid therapy and enhanced hemodynamic monitoring to avoid hypoperfusion, a key driver of AKI and liver injury (15). Promote the use of lung-protective ventilation strategies to reduce ARDS risk (16). Given the varied training levels among anesthesiologists in county-level hospitals, we see an urgent need to establish centralized, expert-guided hemodynamic management support systems.

### 3.3 Tertiary prevention (postoperative)

Establish a “Surgical Rescue” team trained in the early recognition and management of POI. Implement standardized care pathways for AKI (e.g., avoiding nephrotoxins) and delirium (e.g., early mobilization, sleep hygiene). To address relative staff shortages, the development and use of intelligent clinical support systems for monitoring and alerting are needed.

According to the findings of global ICU needs assessment surveys, there is significant variation in ICU resources and staffing worldwide, which may lead to differences in practices and outcomes across healthcare systems (17). ICU resources in Southeast and South Asia are often inadequate in terms of bed availability, equipment, and the number of specialized professionals. For instance, in Southeast Asia, the prevalence of ventilator-associated pneumonia (VAP) ranges from 16.2% to 74.2%, with mortality rates as high as 30%, partly attributable to resource constraints and variations in the level of care (18). Disparities may still exist between different regions within China (e.g., urban vs. rural), which mirrors situations in countries like India (19).

### 3.4 Southeast and South Asia

ICU care protocols and guidelines in these regions may be less uniform and standardized due to disparities in healthcare resources and training.

### 3.5 China

ICU care protocols and guidelines in China are relatively more unified and standardized. A study on predictors of VAP prevention practices among ICU nurses in Sarawak revealed a gap between nurses' knowledge levels and their self-reported practices (20). In China, Continuing Professional Development (CPD) programs for nurses also aim to align clinical practice with recommended standards (21).

## 4 AI-assisted risk assessment and early warning systems

Digital infrastructure, by integrating advanced Artificial Intelligence (AI) technologies, enables the construction of powerful risk assessment and early warning systems, thereby allowing for intervention before or during the early stages of organ injury. Machine learning-based analysis of serum creatinine trajectories can identify the risk of AKI in critically ill septic patients earlier and more accurately, as it captures the dynamics and inherent complexity of creatinine changes, not just peak creatinine levels (22). AI has also demonstrated significant advantages in predicting postoperative complications such as cardiovascular events (23). Beyond threshold-based alerts, systems can utilize AI algorithms to identify potential risk patterns, such as early trends in hemodynamic instability or dynamic changes in infection markers, and send timely warnings to healthcare staff to support clinical decision-making.

## 5 Conclusion

The work by Kork et al. provides a powerful evidence base for the world. For China, it serves as a crucial wake-up call. The fight against perioperative mortality and morbidity will be won not only in the high-tech ICUs of metropolitan centers but, more importantly, in the operating rooms and wards of thousands of county hospitals. By prioritizing resource-appropriate, preventative strategies and building a robust perioperative medicine system, we can mitigate this global burden and significantly improve surgical outcomes for the millions of patients we serve.
